# Screening for HPV infection in exfoliated cervical cells of women from different ethnic groups in Yili, Xinjiang, China

**DOI:** 10.1038/s41598-019-39790-2

**Published:** 2019-03-05

**Authors:** Zhenzhen Pan, Yuning Song, Xiangyi Zhe, Weibin Wang, Junling Zhu, Weinan Zheng, Hongtao Li, Dongmei Li, Dongdong Cao, Zemin Pan, Renfu Shao

**Affiliations:** 1 Key Laboratory of Xinjiang Endemic and Ethnic Diseases, Department of Biochemistry and Molecular Biology, School of Medicine, Shihezi University, Shihezi, 832002 Xinjiang China; 2 Department of Clinical Laboratory, the Fourth Division Hospital of Xinjiang Production and Construction Corps, Yining, 835000 Xinjiang China; 3 Department of Clinical Laboratory, Friendship Hospital of Yili Kazak Autonomous Region of Xinjiang, Yining, 835000 Xinjiang China; 4 Department of Medicine, the Fourth Division Hospital of Xinjiang Production and Construction Corps, Yining, 835000 Xinjiang China; 5 Department of Pathology, the First People Hospital of Kashgar, Kashgar, 844000 Xinjiang China; 6 School of Science and Engineering, Genecology Research Centre, The Animal Research Centre, University of the Sunshine Coast, Sippy Downs, 4556 Queensland Australia

## Abstract

We investigated the infection status and genotype distribution of human papillomavirus (HPV) in women of different ages and various ethnic groups in the Yili region, Xinjiang, China. We checked the HPV genotypes of 3,445 samples of exfoliated cervical cells using the PCR–reverse dot blot method. The total infection rate of HPV was 25.60% (882/3,445). The ethnic stratification showed that the infection rates were 22.87% (196/857) in Uygur, 21.55% (122/566) in Kazak, and 27.89% (564/2,022) in Han individuals. The most prevalent high-risk genotypes were HPV16, HPV52, and HPV53 in Uygur and Kazak and HPV16, HPV52, and HPV58 in Han ethnic groups. The age stratification showed that the infection rates in Han, Uygur, and Kazak women were up to 40.9% (61/149) in those aged 26–30 years, 41.5% (22/53) in those over 61 years old, and 30.2% (29/96) in those 46–50 years old, respectively. Therefore, HPV infection and HPV genotype distribution varied among the different age groups of the three ethnic groups.

## Introduction

Approximately 527,600 new cervical cancer cases and 265,700 cases of death due to cervical cancer were recorded worldwide in 2012. Cervical cancer is the third most common cause of female cancer deaths in developing countries. The incidence and mortality rates of cervical cancer in low-income countries are higher than those in high-income countries, and approximately 90% of cervical cancer deaths occur in developing countries^1^. In 2015, approximately 98,900 new cases of cervical cancer and 30,500 associated deaths were recorded in China^2^. Xinjiang is one of the areas in China with the highest incidence of cervical cancer. The prevalence of cervical cancer in Uygur women is significantly higher than that in Han and other ethnic groups. One of the highest incidence rates of cervical cancer in Xinjiang has been detected in Uygur women (527/100,000), involving mostly young women^3^. Human papillomavirus (HPV) is the leading cause of cervical cancer. Therefore, cervical cancer can be prevented, discovered in the early stages, and cured. HPV is widespread in humans, and more than 120 types of HPV have been isolated. More than 20 genotypes are classified as high-risk HPV, of which HPV16 and HPV18 are the most carcinogenic^4^. Together, these two genotypes cause ~70% of cervical cancer cases. The key risk factors for HPV carcinogenesis are persistent HPV infection with abnormal expression of HPV early proteins and instability of the HPV gene, resulting in host gene imbalance^5^. The distribution of HPV genotypes in different regions varies widely^6^. The HC2 High-Risk HPV DNA Test, approved by the US Food and Drug Administration (FDA), is a standard method for detecting the 13 high-risk HPV viruses; however, it does not distinguish among specific genotypes^7^. In 2014, the FDA approved the use of a PCR-based genotyping method for first-line screening of cervical cancer in women aged 25 years or older^8^. In 2015, the guidelines set by the Society of Gynecologic Oncology (SGO) and the American Society for Colposcopy and Cervical Pathology (ASCCP) addressed the issue of using HR-HPV testing alone as the primary screening method, and recommended genotyping for HPV16 and 18 as a classification method for HR-HPV-positive women^9^. Recently, Sun *et al*. showed that a PCR-reverse dot blotting HPV test could provide a reliable and sensitive clinical reference^10^. In the current study, we aimed to investigate the distribution of HPV genotypes in different ethnic and age groups in Yili, Xinjiang, China. This study will help us further to understand HPV infection and provide information about the diagnosis and treatment of HPV-related cancers and the development of vaccines in local regions in Xinjiang.

## Results

### HPV infection rate among different ethnic groups

A total of 3,445 samples were analyzed, of which 882 were positive for HPV infection; the infection rate was 25.60% (882/3,445). The HPV infection rates in women of Han, Uygur, and Kazak ethnicities were 27.89% (564/2,022), 22.87% (196/857) and 21.55% (122/566), respectively. The HPV infection rate was significantly different among the three ethnic groups (χ^2^ = 13.797, *P* = 0.001) (Table 1). There was a statistically significant difference between each pair of groups in the Bonferroni step-down procedure. The difference between the Han and the Uygur test results was statistically significant, as was the difference between the Han and the Kazak test results. There was no significant difference between the results of the Uygur and the Kazak groups.

**Table 1:** HPV infection rates of different ethnic groups.

Ethnic groups	N	Positive cases	Negative cases	Positive rate (%)	χ^2^	*P* value
Han	2,022	564	1,458	27.89		
Uygur	857	196	661	22.87	13.797	0.001
Kazak	566	122	444	21.55		
Total	3,445	882	2,563	25.60		

### The most common HPV genotypes in different ethnic groups

The most common high-risk genotype was HPV16, with an overall infection rate of 7.34% (253/3,445), followed by HPV52 with an infection rate of 4.06% (140/3,445) and HPV53 with an infection rate of 2.67% (92/3,445). The most common high-risk genotypes in the Han sample were HPV16, with an infection rate of 18.04% (140/776), HPV52, with an infection rate of 12.89% (100/776), and HPV58, with an infection rate of 7.09% (55/776). In the Uygur women, the most common high-risk genotypes were HPV16, with an infection rate of 25.19% (67/266), HPV52, with an infection rate of 9.40% (25/266), and HPV53, with an infection rate of 6.77% (18/266). The most common high-risk genotypes in the Kazak individuals were HPV16, with an infection rate of 24.08% (46/191), HPV53, with an infection rate of 10.99% (21/191), and HPV52, with an infection rate of 7.85% (15/191). The HPV genotype composition varied among the three ethnic groups, and HPV16 infection differed significantly among the three ethnic groups (χ^2^ = 7.965, *P* = 0.019; Table 2).

**Table 2:** Proportion of HPV genotypes in different ethnic groups.

HPV types	Positive composition ratio N (%)		
	Han	Uygur	Kazak
HPV6	26(3.35)	5 (1.88)	5 (2.62)
HPV11	9 (1.16)	1 (0.38)	7 (3.66)
HPV16	140 (18.04)	67 (25.19)	46 (24.08)
HPV18	20 (2.58)	7 (2.63)	2 (1.05)
HPV31	31 (3.99)	14 (5.26)	5 (2.62)
HPV33	32 (4.12)	3 (1.13)	2 (1.05)
HPV35	9 (1.16)	8 (3.01)	5 (2.62)
HPV39	14 (1.80)	5 (1.88)	6 (3.14)
HPV42	34 (4.38)	17 (6.39)	8 (4.19)
HPV43	27 (3.48)	9 (3.38)	7 (3.66)
HPV45	6 (0.77)	4 (1.50)	0 (0.00)
HPV51	44 (5.67)	8 (3.01)	7 (3.66)
HPV52	100 (12.89)	25 (9.40)	15 (7.85)
HPV53	53 (6.83)	18 (6.77)	21 (10.99)
HPV56	31 (3.99)	4 (1.50)	8 (4.19)
HPV58	55 (7.09)	12 (4.51)	8 (4.19)
HPV59	20 (2.58)	14 (5.26)	8 (4.19)
HPV66	31 (3.99)	16 (6.02)	8 (4.19)
HPV68	28 (3.61)	3 (1.13)	3 (1.57)
HPV73	6 (0.77)	8 (3.01)	4 (2.09)
HPV81	52 (6.70)	13 (4.89)	14 (7.33)
HPV82	5 (0.64)	4 (1.50)	2 (1.05)
HPV83	3 (0.39)	1 (0.38)	0 (0.00)
Total	776 (100)	266 (100)	191 (100)

### Infection with multiple HPV genotypes in different ethnic groups

Overall, the rate of infection with a single HPV genotype was 71.31% (629/882). The double infection rate, i.e. infection with two HPV genotypes, was 21.43% (189/882); the triple infection rate was 5.10% (45/882), and the rate for four or more infections was 1.7% (19/882). According to ethnic group, the rates of single infection, double infection, triple infection, and quadruple or more infection were 72.87% (411/564), 20.57% (116/564), 4.96% (28/564), and 1.59% (9/564) in the Han women; 71.94% (141/196), 21.43% (42/196), 4.59% (9/196), and 2.04% (4/196) in the Uygur women; and 63.11% (77/122), 25.41% (31/122), 6.56% (8/122), and 4.92% (6/122) in the Kazak women. Infection with a single HPV genotype was most common among all three ethnic groups (Table 3).

**Table 3:** Composition ratios of multiple HPV infections in different ethnic groups.

Infection type	Han		Uygur		Kazak		N	Total composition ratio (%)
	N	Composition ratio (%)	N	Composition ratio (%)	N	Composition ratio (%)		
single	411	72.87	141	71.94	77	63.11	629	71.32
secondary	116	20.56	42	21.43	31	25.41	189	21.43
triple	28	4.96	9	4.59	8	6.56	45	5.10
quadruple and over quadruple	9	15.96	4	2.04	6	4.92	19	2.15
Total	564	100	196	100	122	100	882	100

### HPV infection in different age groups of the three ethnic groups

Overall, the HPV infection rates were 34.07% (93/273) for the group aged 26–30 years and 31.3% (36/115) for the group aged ≤25 years. In the Han women, the HPV infection rates were 40.94% (61/149) for the group aged 26–30 years and 36.00% (27/75) for the group aged 56–60 years. In the Uygur women, the HPV infection rates were 33.87% (21/62) for the group aged 26–30 years and 41.51% (22/53) for the group aged ≥61 years. In the Kazak women, the HPV infection rates were 30.21% (14/53) in the group aged 46–50 years and 25.00% (5/20) in the group aged ≤25 years. The HPV infection rates differed significantly among the three ethnic groups aged 26–30 years (χ^2^ = 10.491, *P = *0.005) and ≥61 years (χ^2^ = 9.915, *P* = 0.007) (Table 4).

**Table 4:** HPV infection rates of different age groups among three ethnic groups.

Age groups (years)	Han				Uygur					Kazak			Total positive rate (%)
	N	Positives cases	Negative Cases	Positive rate (%)	N	Positives cases	Negative Cases	Positive rate (%)	N	Positives cases	Negative Cases	Positive rate (%)	
≤25	67	22	45	32.84	28	9	19	32.14	20	5	15	25.00	31.30
26–30	149	61	88	40.94	62	21	41	33.87	62	11	51	17.74	34.07
31–35	189	59	130	31.22	94	16	78	17.02	69	13	56	18.84	25.00
36–40	258	67	191	25.97	132	21	111	15.91	105	15	90	14.29	20.81
41–45	419	114	305	27.21	175	39	136	22.29	89	19	70	21.35	25.18
46–50	473	122	351	25.79	184	34	150	18.48	96	29	67	30.21	24.57
51–55	240	60	180	25.00	88	26	62	29.55	53	14	39	26.42	26.25
56–60	75	27	48	36.00	41	8	33	19.51	20	6	14	30.00	30.15
≥61	152	32	120	21.05	53	22	31	41.51	52	10	42	19.23	24.90
Total	2,022	564	1458	27.89	857	196	661	22.87	566	122	444	21.55	25.60

### HPV infection status in different ethnic groups

Of the 3,445 participants, 55 had cervical cancer; the prevalence rate was 1.60% (55/3,445). Of these 55 cancer patients, 21 were Han women, 20 were Uygur women and 14 were Kazak women. The prevalence rate was 1.04% (21/2,022) for Han women, 2.33% (20/857) for Uygur women and 2.47% (14/566) for Kazak women. Thirteen of the 55 patients (23.64%) were negative for HPV infection, and the other 42 (76.36%) were HPV positive. Of these 42 HPV-positive patients, 19 were Han, 14 Uygur and 9 Kazak. Furthermore, 14 of the 19 Han patients (73.68%) were HPV16 positive; 12 of the 14 Uygur patients (85.71%) were HPV16 positive; and 8 of the 9 Kazak patients (88.89%) were HPV16 positive. Of the 55 patients, 30 (54.55%) had single HPV infection; 10 (18.18%) had double HPV infection; and 2 (3.64%) had triple HPV infection. No quadruple or more than quadruple HPV infection was observed in these patients (Table 5).

**Table 5:** HPV infection status of cervical cancer cases among different ethnic groups.

Ethnic groups	cervical cancer N	HPV-positive cases	HPV Negative cases	HPV-positive rate (%)	HPV16 Positive cases	HPV16 Negative cases	HPV16 Positive rate (%)
Han	21	19	2	90.48	14	5	73.68
Uygur	20	14	6	70.00	12	2	85.71
Kazak	14	9	5	64.29	8	1	88.89
Total	55	42	13	76.36	34	8	80.95

### Relationship between HPV and thin-prep cytologic test (TCT)

Among the 814 patients who were simultaneously tested for HPV and by TCT, 147 patients (18.06%) had HPV infection. In 30 cases of cervical squamous cell carcinoma by TCT diagnosis, 24 (80.00%) had HPV infection. In 756 cases of cervical intraepithelial neoplasia (CIN), 118 (15.61%) had HPV infection. In 28 cases of cervical adenocarcinoma and polyps, 5 (17.86%) had HPV infection. A statistically significant difference was observed among the groups (χ^2^ = 112.39, *P* = 0.000) (Table 6).

**Table 6:** Relationship between HPV infection and TCT.

TCT	N	HPV-positive cases	HPV-positive rate (%)	χ^2^	*P*
CIN	756	118	15.61		
Cervical squamous cell carcinoma	30	24	80.00	112.39	0.000
Others (adenocarcinoma and polyps)	28	5	17.86		
Total	814	147	18.06		

### Relationship of HPV infection with pregnancy and multiparous women

A total of 814 patients were simultaneously tested for HPV infection and by TCT. The HPV infection rate of G0P0–G5P5 patients was lower than the average infection rate of 18.06% (147/814). The HPV infection rate of G6P0–G10P10 patients was higher than the average infection rate of 18.06% (147/814). A significant difference was observed between the groups (χ^2^ = 26.03, *P* = 0.001) (Table 7).

**Table 7:** Relationship between HPV infection and pregnant and multiparous women.

Pregnant and multiparous women	N	HPV-positive cases	HPV-positive rate (%)	χ^2^	*P*
G0P0	20	3	15.00		
G1P0-G1P1	109	14	12.84		
G2P0-G2P2	233	39	16.74		
G3P0-G3P3	186	33	17.74		
G4P0-G4P4	129	23	17.83	26.032	0.001
G5P0-G5P5	69	10	14.49		
G6P0-G6P6	38	11	28.90		
G7P0-G7P7	18	8	44.40		
G8P0-G10P10	12	6	50.00		
Total	814	147	18.06		

## Discussion

HPV infection is sexually transmitted and closely related to the occurrence of cervical cancer^11^. It is also a direct cause of cervical cancer. Therefore, HPV screening is essential for the early diagnosis and prevention of cervical cancer. Overall, the HPV-positive rate is 20–46% in human populations^12,13^. Our survey showed that the rate of HPV infection was 27.1% in Yili, which is in the western part of Xinjiang, China. The rates of HPV infection were 29.3% in Han women, 24.7% in Uygur women, and 21.7% in Kazak women. The rates of HPV infection among these three ethnic groups were substantially different. In the resident population of Yili, 47% are of Uygur ethnicity, 36% are Han, and 4.7% are Kazak. Of the 3,445 participants, only 25% of the participants were of Uygur women; this may be related to health awareness and economic level. The risk of cervical cancer is considerably higher in women who have undergone multiple full-term pregnancies than in nulliparous women^14^. Our survey showed that the HPV infection rate was higher in pregnant and multiparous women than that in non-pregnant and less fecund women. In contrast to Han people, Uygur and Kazak people have similar lifestyles and living habits, such as earlier marrying age, earlier age at first pregnancy, and having more pregnancies and maternal deliveries. These lifestyles and living habits may be related to the higher incidence of cervical cancer in Uygur and Kazak women. In general, Uygur women have a higher divorce rate, whereas Kazak women have a lower divorce rate than Han women and the national average rate.

This study revealed that three high-risk HPV genotypes, namely HPV16, HPV52, and HPV53, had higher rates than other genotypes in Yili. The most prevalent high-risk HPV genotypes in Han women were HPV16, HPV52, and HPV58. The most prevalent high-risk HPV genotypes in both Uygur and Kazak women were HPV16, HPV52, and HPV53. The HPV16 infection rate in Uygur women was remarkably higher than that in Han and Kazak women. HPV16 is the most common high-risk genotype associated with cervical cancer. The higher incidence of cervical cancer in Uygur women may be associated with their higher HPV16 infection rate in comparison with other ethnic groups. HPV infection rates vary regionally in China. For instance, in Beijing, the HPV infection rate is 19.1%, and the most common genotypes are HPV52, HPV16, and HPV58^15^. In Shenzhen, the HPV infection rate is 15.9%, and the most common genotypes are HPV52, HPV16, and HPV53^16^. In Kashi, Xinjiang, the HPV infection rate is 13%, and the most common genotypes are HPV16, HPV58, and HPV39^17^. In Uygur women, the HPV infection rate is 20.8%, and the most common genotypes are HPV16, HPV31, HPV51, HPV56, and HPV39^18^. Although the HPV infection rate and the most common genotypes vary among regions and among ethnic groups, HPV16 is one of the most common genotypes in all studies.

Our age stratification results showed that the HPV infection rate in Han, Uygur, and Kazak women was higher in the group aged ≤30 years than in other age groups. This finding may be related to active sexual behavior and the peak stage of marriage and pregnancy. The HPV infection rate decreased as the individuals aged. Approximately 80% of sexually active women are infected by at least one HPV genotype in their lives, but most of the HPV infections are transient. HPV can be cleared by the autoimmune system within 2 years, and only approximately 10% of persistent HPV infections last 5–10 years and progress from low-grade to high-grade cervical intraepithelial neoplasia, eventually developing into cervical cancer^4^. The HPV infection rate in Uygur women reached 41.51% in the group aged ≥61 years, which was considerably higher than that in Han and Kazak groups. This result indicated a weaker ability to clear HPV and thus a longer duration of HPV infection in Uygur women than in other ethnic groups. Long-term HPV infection may eventually increase the incidence of cervical cancer and may decrease the function of the immune system because of changes in hormone levels or the effect of HPV in Uygur women^19,20^. The present study showed that the prevalence of cervical cancer in Kazak women was also higher than that in Han women, but the reason is unknown.

This research examined 17 types of high-risk HPV and 6 types of low-risk HPV using a PCR-dot reverse hybridization assay and further confirmed the cervical disease by a thin-prep cytological test. We found that the rate of cervical cancer associated with HPV infection was considerably higher when compared with CIN. However, some cancer patients had negative tests for HPV infection, possibly because more than 10 other types of high-risk HPV were not assayed in this study or because their cervical cancer had a genetic origin.

Cervical cancer vaccines have been used in some developed countries. However, they are not used widely in underdeveloped countries and regions, including China, because of the high cost of the vaccines. Semicompulsory vaccination of cervical cancer has been proposed in China^21^. Before HPV vaccines become widely used in China, HPV detection will remain an important means to prevent and control cervical cancer. In summary, HPV infection varies among different regions and ethnic groups. Vaccine design should address the common genotypes to achieve the best prevention outcomes.

## Conclusion

The HPV infection rates differed among Han, Uygur, and Kazak women in Yili in Xinjiang. The HPV genotype distribution also varied among ethnic and age groups.

## Materials and Methods

### Sample collection

With informed consent from the participants, samples of cervical mucus from 3,445 women were examined from 2015 to 2017 in Yili, Xinjiang, China. The participants were from 17 to 86 years old, and their median age was 44 years. Among them, 857 were of Uygur ethnicity, 566 were Kazak, and 2,022 were Han. All 3,445 participants were screened for HPV genotypes. The sample collection for this study was approved by the Ethical Committee of the First Affiliated Hospital of the Medical College of Shihezi University, and informed consent was obtained from all of the participants. Informed consent was obtained from the parents of participants who were 17 years old. The sampling was carried out in accordance with the approved guidelines by gynecologists after a brief initial examination. A vaginal opening device was used to expose the cervix, cotton swabs were employed to wipe off the excess cervical secretions, and an exfoliated cervical cell collector was utilized for sampling. A cervical brush was placed in the cervix in a single direction of rotation 4–5 times to obtain sufficient epithelial cell samples. Next, the head of the cervical brush was placed in an elution tube, the brush handle broken off, and the elution tube cover was tightened immediately. Samples that could not be immediately tested to detect the genotype were stored in a refrigerator at 4 °C for 7 days.

### PCR-reverse dot hybridization assay

The HPV genotyping (23 types) was performed with PCR-reverse dot blot hybridization technology (Shenzhen Co., Ltd., China). All of the detection procedures were conducted in accordance with the manufacturer’s instructions. Detection involved HPV DNA extraction, PCR amplification, hybridization, membrane washing, color development, compliance with quality standards, and data interpretation. With this method, 23 different HPV genotypes could be detected: 17 high-risk genotypes, HPV16, 18, 31, 33, 35, 39, 45, 51, 52,53, 56, 58, 59, 66, 68, 73 and 82; and 6 low-risk genotypes, HPV6, 11, 42, 43, 81 and 83. The lower limit of the virus copy number of detections was less than 1.0 × 10^4^ copies/ml.

### Thin-prep cytologic test

A gynecologist swabbed the cervical cells, collected them in a tube, and sent them to our laboratory for testing as soon as possible. A pathologist reported the cytology test results^22^.

### Statistical analysis

Data were statistically analyzed with SPSS 17.0. Data were divided into the three different ethnic groups: Uygur, Kazak, and Han. The age group categories were ≤25, 26–30, 31–35, 36–40, 41–45, 46–50, 51–55, 56–60, and ≥61 years. The chi-squared test was used, and *P* < 0.05 was considered statistically significant. The Bonferroni step-down procedure was used to compare two groups to minimize the risk of inflated type 1 error.
